# Development of Store-Operated Calcium Entry-Targeted Compounds in Cancer

**DOI:** 10.3389/fphar.2021.688244

**Published:** 2021-05-28

**Authors:** Xiaojing Liang, Ningxia Zhang, Hongming Pan, Jiansheng Xie, Weidong Han

**Affiliations:** ^1^ Department of Medical Oncology, Sir Run Run Shaw Hospital, College of Medicine, Zhejiang University, Hangzhou, China; ^2^ Laboratory of Cancer Biology, Institute of Clinical Science, Sir Run Run Shaw Hospital, College of Medicine, Zhejiang University, Hangzhou, China

**Keywords:** SOCE, cancer, inhibitor, pharmacodynamics, therapy

## Abstract

Store-operated Ca^2+^ entry (SOCE) is the major pathway of Ca^2+^ entry in mammalian cells, and regulates a variety of cellular functions including proliferation, motility, apoptosis, and death. Accumulating evidence has indicated that augmented SOCE is related to the generation and development of cancer, including tumor formation, proliferation, angiogenesis, metastasis, and antitumor immunity. Therefore, the development of compounds targeting SOCE has been proposed as a potential and effective strategy for use in cancer therapy. In this review, we summarize the current research on SOCE inhibitors and blockers, discuss their effects and possible mechanisms of action in cancer therapy, and induce a new perspective on the treatment of cancer.

## Introduction

### The Overview of Store-Operated Calcium Entry

Store-operated calcium entry (SOCE) is typically activated by ligands of cell surface receptors such as G proteins that activate phospholipase C (PLC) to cleave phosphatidylinositol 4, 5-bisphosphate (PIP2) and produce inositol 1, 4, 5-trisphosphate (IP3). IP3 binds to IP3 receptors (IP3R) on the endoplasmic reticulum (ER) membrane, leading to the release of Ca^2+^ from the ER. Cells respond to this depletion of ER intraluminal Ca^2+^ by opening Ca^2+^ channels to allow cellular influx, which is also the provenance of “SOCE.” Researchers have been exploring the mechanisms and functions of SOCE since its discovery and definition, and it has been found that stromal interaction molecules (STIMs) and ORAI family proteins are the two major participants in SOCE (Hogan et al., 2010; Prakriya and Lewis, 2015).

STIM proteins (STIM1 and STIM2) are located on the ER membrane and are essential for SOCE (Williams et al., 2001; Liou et al., 2005; Roos et al., 2005). STIM1 contains an ER-luminal portion (containing two EF-hands and a sterile α-motif (SAM) domain), a single transmembrane segment, and a cytoplasmic portion (containing a coiled-coil domain (CCD), a STIM-ORAI-activating region (SOAR) or CRAC activation domain (CAD), a serine- or proline-rich segments and a polybasic (PB) C-tail) (Hogan et al., 2010; Xie et al., 2016). STIM2 has a structure similar to STIM1 but a critical residue difference in the SOAR domain, which endows STIM2 with partial agonist properties and competitive inhibiting functions in STIM1-mediated Ca^2+^ entry, thereby maintaining basal cytoplasmic Ca^2+^ levels by preventing uncontrolled activation of ORAI proteins (Brandman et al., 2007; Wang et al., 2014).

ORAI proteins are plasma membrane (PM) channels that can be gated by STIMs for Ca^2+^ entry during SOCE. Three homologs of ORAI have been identified in humans, namely, ORAI1, ORAI2, and ORAI3 (also called CRACM1-3), among which, ORAI1 is the most potent and has been extensively studied. ORAI1 contains four transmembrane helices (TM1-TM4), an intracellular location domain (including N-terminal and C-terminal epitope tags) and an extracellular location domain (including an epitope tag introduced into the TM3-TM4 loop). The N-terminus and C-terminus of ORAI1 intracellular sites are essential for the interaction with STIM1 and the opening of the ORAI1 channel (Peinelt et al., 2006; Vig et al., 2006; Hogan et al., 2010; Xie et al., 2016). The homologs, ORAI2 and ORAI3 primarily differ in cytosolic N-terminal, C-terminal and 3–4 loop sequences (Amcheslavsky et al., 2015), and they mediate SOCE in a manner similar to that of ORAI1, but they differ in permeability properties and inactivation (Lis et al., 2007), leading to augmented SOCE efficacy in the order ORAI1 > ORAI2 > ORAI3 (Mercer et al., 2006).

**FIGURE 1:**
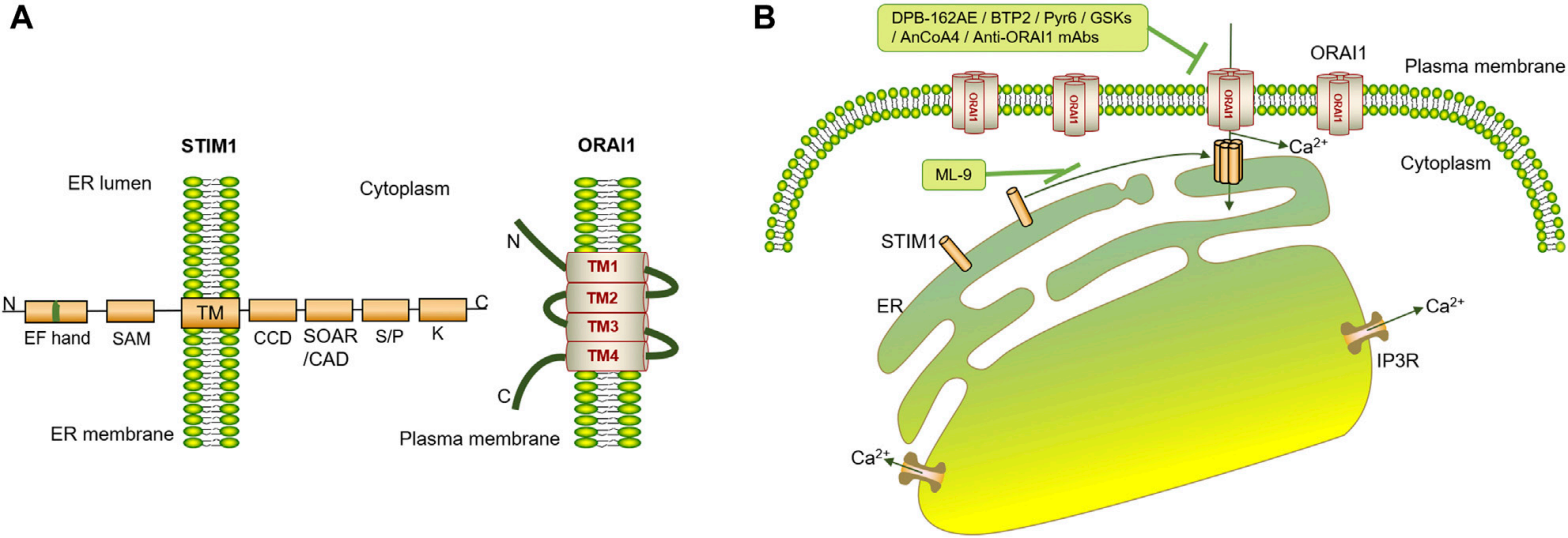
Schematic diagram of ORAI1- and STIM1-mediated SOCE. **(A)** STIM1 contains two EF-hands, a sterile α motif (SAM) domain, a single transmembrane segment, a coiled-coil domain (CCD), a STIM-ORAI activating region (SOAR) or CRAC activation domain (CAD), a serine- or proline-rich segments, and a polybasic (PB) C-tail. ORAI1 contains four transmembrane helices (TM1-TM4), intracellular N-terminal and C-terminal epitope tags, and an extracellular epitope tag that is introduced into the TM3-TM4 loop. **(B)** IP3R binds to IP3 upon triggering by agonists, leading to the release of Ca^2+^ from the ER. STIM1 residing in the ER senses the Ca^2+^ decrease in the ER and initiates conformational changes and oligomerization to expose SOAR/CAD and the PB C-tail. Activated STIM1 further multimerizes and migrates toward the PM, interacts with the intracellular regions of ORAI1, gates and opens ORAI1 directly to enable rapidly uptake of Ca^2+^. SOCE-targeted inhibitors, such as DPB-162AE, BTP2, Pyr6, GSKs, AnCoA4, Anti-ORAI1 mAbs and so on, are demonstrated to inhibit ORAI1, and ML-9 is confirmed to inhibit STIM1 translocation.

When STIM1 residing in the ER lumen senses a Ca^2+^ decrease in the ER, it initiates conformational changes and oligomerization, overcoming the intracellular autoinhibition mediated by CC1 to expose SOAR/CAD and the PB C-tail. Activated STIM1 multimerizes and migrates toward the PM, where it interacts with the intracellular regions of ORAI1, resulting in ORAI1 activation and Ca^2+^ influx (Figure 1) (Liou et al., 2005; Park et al., 2009; Yuan et al., 2009; Zhou et al., 2010).

Transient receptor potential proteins (TRPs) contain similar structures, consisting of six transmembrane-helical domains (TM1-TM6) with a loop between TM5 and TM6 and cytoplasmic N- and C-termini (Venkatachalam and Montell, 2007). TRP channels, especially TRPC channels, have been proposed as candidate components of SOCE, although this assignment is still disputed, under certain conditions, several TRPC channels can function in SOCE pathways (Prakriya and Lewis, 2015). For example, in HEK-293 cells, the knockdown of TRPC1, TRPC3 or TRPC7 dramatically reduced the SOCE activated by passive Ca^2+^ store depletion, while inhibition of TRPC4 or TRPC6 had no effect on SOCs activity (Zagranichnaya et al., 2005). However, in human corneal epithelial cells, mouse endothelial cells and mouse mesangial cells, TRPC4 deficiency decreased SOCE (Tiruppathi et al., 2002; Wang et al., 2004; Yang et al., 2005). Furthermore, TRPC3 and TRPC7 effects on SOCE depend on their expression levels. At low expression levels, they are activated by passive Ca^2+^ store depletion and act as SOCE, while at high expression levels, they behave as Ca^2+^ store-independent Ca^2+^ influx channels (Worley et al., 2007). Therefore, it is necessary to continue to explore the function of TRPCs in SOCE.

### Role of Store-Operated Calcium Entry in Cancer

Accumulating evidence has revealed that many cancers, such as breast, liver, lung, gastric, colon, and ovarian cancer, exhibit augmented SOCE and overexpression of STIM1 or ORAI1. SOCE inhibition through STIM1 or ORAI1 knockdown inhibits the proliferation and metastasis of cancer cells, suggesting that SOCE may act as an oncogenic pathway (Yang et al., 2009; Chen et al., 2011; Yoshida et al., 2012; Motiani et al., 2013; Yang et al., 2013; Zhang et al., 2013; Kim et al., 2014; Umemura et al., 2014; Wang et al., 2015; Xu et al., 2015; Schmid et al., 2016; Xia et al., 2016; Goswamee et al., 2018; Wang Y. et al., 2018; Zang et al., 2019; Huang et al., 2020). In addition, SOCE is believed to promote tumor angiogenesis through the increased secretion of VEGF by endothelial and cancer cells. For example, in cervical cancer, STIM1 regulates the production of VEGF to control the formation of blood vessels, and the formation of tumor could be impaired by inhibiting STIM1 (Chen et al., 2011). In addition, the potential therapeutic value of targeting SOCE in cancer has further been supported by the fact that STIM- and ORAI- mediated SOCE is essential for the secretion of cytokines and chemokines from T cells, mast cells, and macrophages, as well as in the differentiation and functions of CD4^+^ and CD8^+^ T cells. For example, the inhibition of SOCE by genetic deletion of ORAI1, STIM1, or STIM2 in murine CD4^+^ T cells impaired Th17 cell function, causing a decrease in the production of IL-17, which is believed to be proinflammatory factor important for tumor progression (Ma et al., 2010; Shaw et al., 2014; Mehrotra et al., 2019). Moreover, as candidate components of SOCE, TRP channels have been found to affect the survival, proliferation, and invasion of cancer cells (Shapovalov et al., 2016). For example, TRPC1 was confirmed to play different roles in tumorigenesis, inhibition of TRPC1 by siRNA or SOCE inhibitors could suppress the proliferation and invasion of cancer cells including nasopharyngeal carcinoma, malignant glioma and non-small-cell lung carcinoma (Bomben and Sontheimer, 2010; He et al., 2012; Tajeddine and Gailly, 2012).

In summary, SOCE promotes the proliferation and metastasis of cancer cells, and enhances tumor angiogenesis and the formation of a tumor-promoting inflammatory environment. Inhibition of SOCE may be a potential and effective therapy for cancers. In this review, we summarize the available inhibitors of SOCE (Tables 1, 2), discuss their effects and possible mechanisms (Table 3), and introduce a new viewpoint on the treatment of cancer.

**TABLE 1:** Structures and IC_50_ of compounds.

Compound	Structure	IC_50_ of SOCE inhibition	References
SKF96365	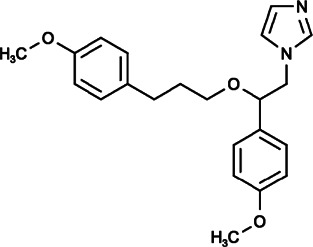	IC_50_ = 10.2 ± 1.2 μM in VSMCs	Zhang et al. (1999)
		IC_50_ = 12 μM in Jurkat T cells	Chung et al. (1994)
		IC_50_ = 1.2 μM in arterioles	McGahon et al. (2012)
		IC_50_ = 60 μM in PLP-B lymphocyte cells	Dago et al. (2018)
2-APB	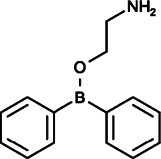	IC_50_ = 4.8 ± 0.6 μM in DT40 cells	Goto et al. (2010)
		IC_50_ = 3.7 μM as inhibitor, >100 μM as activator in arterioles	McGahon et al. (2012)
		IC_50_ = 3 μM in CHO cells	Ozaki et al. (2013)
		IC_50_ = 17 ± 1 μM in human platelets	Giambelluca and Gende (2007)
		IC_50_ = 15 μM in Jurkat T cells	Suzuki et al. (2010)
DPB-162AE	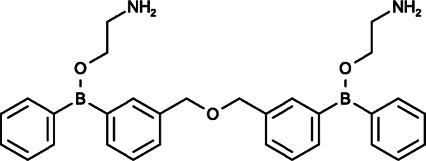	IC_50_ = 27 ± 2 nM in DT40 cells	Goto et al. (2010)
		IC_50_ = 190 ± 6 nM in CHO cells	Goto et al. (2010)
		IC_50_ = 200 nM in HEK293 cells	Hendron et al. (2014)
		IC_50_ = 0.32 μM in Jurkat T cells	Suzuki et al. (2010)
DPB-163AE	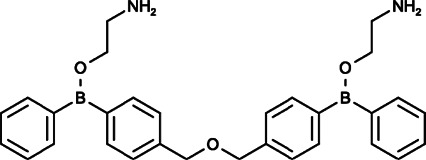	IC_50_ = 42 ± 3 nM in DT40 cells	Goto et al. (2010)
		IC_50_ = 210 ± 20 nM in CHO cells	Goto et al. (2010)
		IC_50_ = 600 nM in HEK293 cells	Hendron et al. (2014)
		IC_50_ = 0.43 μM in Jurkat T cells	Suzuki et al. (2010)
BTP2	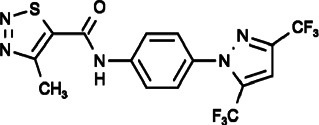	IC_50_ = 100 nM in Jurkat T cells	Ishikawa et al. (2003)
		IC_50_ = ∼10 nM in peripheral blood T-lymphocytes	Zitt et al. (2004)
		IC_50_ = 0.1–0.3 μM in HEK293 cells, DT40 B cells and A7r5 smooth muscle cells	He et al. (2005)
		IC_50_ = 0.59 μM in RBL-2H3 cells	Schleifer et al. (2012)
Pyr3	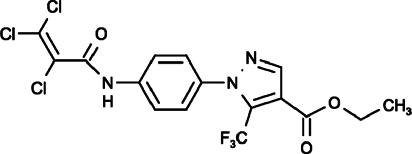	IC_50_ = 0.5 μM in E13 cortical neurons	Gibon et al. (2010)
		IC_50_ = 0.54 μM in RBL-2H3 cells	Schleifer et al. (2012)
Pyr6	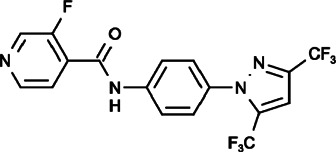	IC_50_ = 0.49 μM in RBL-2H3 cells	Schleifer et al. (2012)
Pyr10	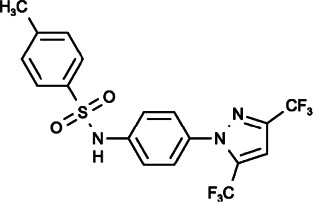	IC_50_ = 13.08 μM in RBL-2H3 cells	Schleifer et al. (2012)
GSK-5498A	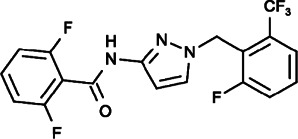	IC_50_ = ∼1 μM in human embryonic kidney cells	Rice et al. (2013)
		IC_50_ = 3.7 μM in ASMCs	Chen and Sanderson (2017)
GSK-5503A	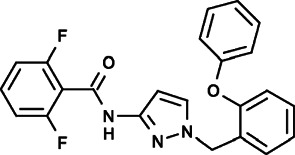	IC_50_ = ∼4 μM in HEK cells	Derler et al. (2013)
GSK-7975A	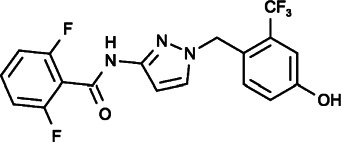	IC_50_ = ∼4 μM in HEK cells	Derler et al. (2013)
		IC_50_ = 4.1 μM in ASMCs	Chen and Sanderson (2017)
		IC_50_ = 0.34 μM in HLMCs	Ashmole et al. (2012)
Synta66	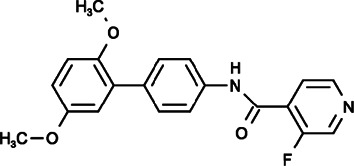	IC_50_ = ∼1 μM in Jurkat T cells	Di Sabatino et al. (2009)
		IC_50_ = 3 μM in RBL-1 mast cells	Ng et al. (2008)
		IC_50_ = 0.25 μM in HLMCs	Ashmole et al. (2012)
CAI	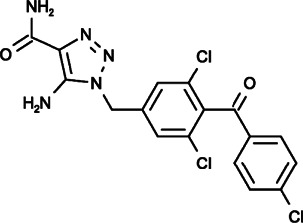	IC_50_ = 2∼5 μM in Huh-7 cells	Enfissi et al. (2004)
CM4620	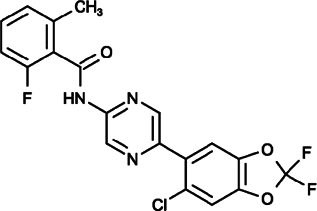	IC_50_ = 120 nM for ORAI1/STIM1-mediated SOCE in HEK 293 cells	Wen et al. (2015); Waldron et al. (2019)
		IC_50_ = 900 nM for ORAI2/STIM1-mediated SOCE in HEK 293 cells	
ML-9	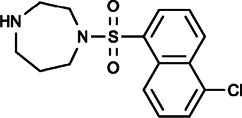	IC_50_ = ∼16 μM in HEK293 cells	Smyth et al. (2008)
NPPB	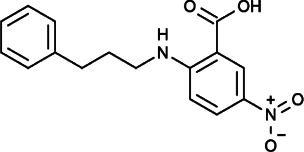	IC_50_ = 5 μM in Jurkat T cells	Li et al. (2000)
AnCoA4	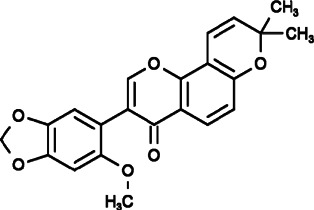	Not found	Not found
RO2959	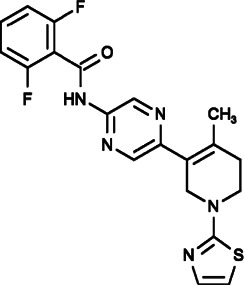	IC_50_ = 402 ± 129 nM in RBL-2H3 cells	Chen G. et al. (2013)
		IC_50_ = 265 ± 16 nM in CD4^+^ T cells	Chen G. et al. (2013)
YZ129	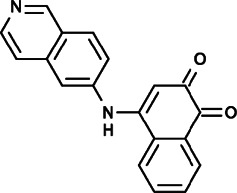	IC_50_ = 820 ± 130 nM in HeLa cells	Liu et al. (2019)
MRS-1845	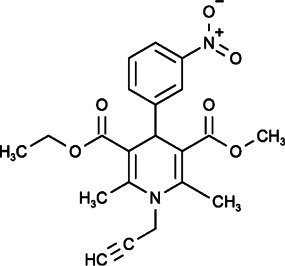	IC_50_ = 1.7 μM in HL-60 cells	Harper et al. (2003)
DES	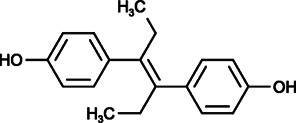	IC_50_ = ∼1 μM in GBM cells	Liu et al. (2011)
Mibefradil	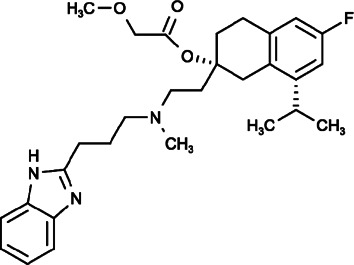	IC_50_ = 52.6, 14.1, and 3.8 μM for ORAI1, ORAI2, and ORAI3 respectively in HEK-293 T-REx cells	Li et al. (2019)

**TABLE 2:** Targeting channels of compounds.

Compound	Channel	Mechanism	References
SKF96365	SOCE	Inhibitor	Iwamuro et al. (1999)
	RMCE	Inhibitor	Merritt et al. (1990); Cabello and Schilling (1993)
	TRP channels	Inhibitor	Bennett et al. (2001)
	CCE	Inhibitor	Wu et al. (1999)
2-APB	SOCE IP3R channel TRP channels	Activator-Low concentration	Peinelt et al. (2008)
		Inhibitor-high concentration inhibitor	Peinelt et al. (2008)
		Activator-TRPV1 TRPV2 TRPV3 TRPM6 TRPC3	Maruyama et al. (1997)
		Inhibitor-TRPC1 TRPC3 TRPC4 TRPC5 TRPC6 TRPC7 TRPM2 TRPM3 TRPM7 TRPM8 TRPV3 TRPV6	Ma et al. (2003); Chung et al. (2004); Hu et al. (2004); Li et al. (2006); Colton and Zhu (2007)
			Hu et al. (2004); Poburko et al. (2004); Li et al. (2006); Colton and Zhu (2007); Chokshi et al. (2012)
DPB-162AE	SOCE	Specific inhibitor	Goto et al. (2010); Hendron et al. (2014); Bittremieux et al. (2017)
DPB-163AE	SOCE	Activator-Low concentration	Goto et al. (2010); Hendron et al. (2014)
		Inhibitor-high contration	Goto et al. (2010); Hendron et al. (2014)
BTP2	SOCE	Inhibitor	Ishikawa et al. (2003)
	CRAC	Inhibitor	Zitt et al. (2004)
	TRPC1	Inhibitor	Oláh et al. (2011)
	TRPC6	Inhibitor	Wu et al. (2017)
	TRPC3	Inhibitor	He et al. (2005)
	TRPC5	Inhibitor	He et al. (2005)
	TRPM4	Activator	Takezawa et al. (2006)
Pyr3	SOCE	Inhibitor	Schleifer et al. (2012)
	TRPC3	Inhibitor	Kiyonaka et al. (2009); Oda et al. (2017); Wang et al. (2019)
Pyr6	SOCE	Inhibitor	Schleifer et al. (2012); Chauvet et al. (2016)
	TRPC3	Inhibitor	Schleifer et al. (2012); Chauvet et al. (2016)
Pyr10	TRPC3	Inhibitor	Schleifer et al. (2012)
GSK (GSK-5503A/GSK-7975A/GSK-5498A)	SOCE	Inhibitor	Yamashita et al. (2007); Derler et al. (2013); Jairaman and Prakriya (2013); Rice et al. (2013); Chen and Sanderson (2017)
	TRPV6	Inhibitor	Owsianik et al. (2006); Jairaman and Prakriya (2013)
Synta66	SOCE	Specific inhibitor	Li et al. (2011)
mAbs	Orai1	Inhibitor	Cox et al. (2013); Lin et al. (2013)
CAI	SOCE	Inhibitor	Sjaastad et al. (1996)
	RMCE	Inhibitor	Hupe et al. (1991); Kohn et al. (1994)
	VDCE	Inhibitor	Hupe et al. (1991); Kohn et al. (1994)
RP4010	SOCE	Inhibitor	Cui et al. (2018)
CM4620	SOCE	Inhibitor	Wen et al. (2015)
ML-9	Myosin light chain kinases	Inhibitor	Saitoh et al. (1987)
	SOCE	Inhibitor	Smyth et al. (2008); Cui et al. (2017)
	CRAC	Inhibitor	Smyth et al. (2008)
	TRPC5	Inhibitor	Shimizu et al. (2006)
NPPB	CCE	Inhibitor	Gericke et al. (1994)
	CRAC	Inhibitor	Li et al. (2000)
AnCoA4	SOCE	Inhibitor	Sadaghiani et al. (2014)
RO2959	SOCE	Inhibitor	Chen G. et al. (2013)
YZ129	NFAT	Inhibitor	Liu et al. (2019)
MRS-1844/MRS-1845	SOCE	Inhibitor	Harper et al. (2003); van Kruchten et al. (2012)
	L-type channels	Inhibitor	Harper et al. (2003)
DES	SOCE	Inhibitor	Zakharov et al. (2004); Ohana et al. (2009); Dobrydneva et al. (2010)
NSAIDs	SOCE	Inhibitor	Núñez et al. (2006)
Mibefradil	SOCE	Inhibitor	Li et al. (2019)

**TABLE 3:** SOCE inhibitors in cancer research.

Compound	Cancer type	Cell lines	Major effect	Possible mechanisms	References
SKF96365	MG astrocytoma	U373	Inhibition of cell growth	Promotes the accumulation of [3H]IP1 and inhibits the mobilization of intracellular Ca^2+^	Lee et al. (1993); Arias-Montaño et al. (1998)
	Cervical cancer	Hela	Inhibition of cell growth and cell migration	Promotes the accumulation of [3H]IP1	Arias-Montaño et al. (1998); Chen Y.-T. et al. (2013)
		SiHa		Inhibits the activation of non-muscle myosin II and the formation of actomyosin via blocking SOCE channel	
	Neuroblastoma	SK-N-MC	Inhibition of cell growth	Inhibits the mobilization of intracellular Ca^2+^	Lee et al. (1993)
	Gastric cancer	AGS and MKN45	Inhibition of cell growth and tumor formation	Arrests cell cycle in G2/M phase	Cai et al. (2009)
	Nasopharyngeal carcinoma	CNE2 and HONE1	Inhibition of cell proliferation and colony formation, promotion of apoptosis and cell-cycle	Distracts the transduction of oncogenic Ca^2+^ signaling	Zhang et al. (2015)
	Colorectal cancer	HCT116 and HT29	Inhibition of cell growth and tumor formation, inducement of apoptosis, cell-cycle and autophagy	Inhibits the calcium/CaMKIIγ/AKT-mediated pathway	Jing et al. (2016)
	Prostate cancer	DU145 and PC3	Inhibition of the survival and proliferation	Inhibits AKT/mTOR pathway and induces autophagy	Selvaraj et al. (2016)
	Multiple myeloma	KM3 and U266	Inhibition of cell viability and proliferation, inducement of apoptosis	Arrests cell cycle in G2/M phase. Induces the reduction of [Ca^2+^]i	Wang W. et al. (2018)
	Ovarian cancer	SKOV3	Inhibition of cell proliferation	Arrests cell cycle in G2/M phase through inhibiting TRPC channels	Zeng et al. (2013)
	Glioma and glioblastoma	U251, U87, D54MG, LN-229, T98G, U373	Inhibition of cell growth and colony formation. Increase in the sensitivity to irradiation	Inhibits TRPC channels. Arrests cell cycle in G2/M phase	Bomben and Sontheimer (2008); Ding et al. (2010); Song et al. (2014)
	Breast tumor	MDA-MB-231	Inhibition of cell migration, inhibition of tumor Metastasis in mice	Impairs the assembly and disassembly of focal adhesions	Yang et al. (2009)
	Melanoma	WM793	Inhibition of cell invasion	Blocks the formation, activity of invadopodium	Sun et al. (2014)
	Non-small cell lung cancer	A549	Inhibition of cell migration and proliferation	Inhibits TRPC channels and SOCE channel	Wang Y. et al. (2018)
2-APB	Rhabdomyosarcoma (RMS)	RD, RH30	Inhibition of cell proliferation, migration and invasion	Inhibits SOCE channel	Schmid et al. (2016)
	Melanoma	WM793	Inhibition of cell invasion and lung metastasis	Blocks the formation, activity and maturation of invadopodium	Sun et al. (2014)
	Cervical cancer	SiHa	Inhibition of cell migration	Inhibits actomyosin formation and cellular contractile force via blocking SOCE channel	Chen Y.-T. et al. (2013)
	Ovarian cancer	SKOV3	Inhibition of cell proliferation	Inhibits TRPC channels	Zeng et al. (2013)
	Breast cancer	MDA-MB-231, AU565, T47D	Inhibition of cell viability and proliferation	Arrests cell cycle in S phase	Liu et al. (2020)
	Prostate cancer	DU145, PC3	Inhibition of cell migration	Inhibits EMT via blocking TRPM7 channel activity	Sun et al. (2018)
	Glioma and glioblastoma	D54MG, U87	Inhibition of cell development, migration and invasion	Inhibits SOCE channel and TRPC channels (TRPC1, TRPC3, TRPC5, TRPC6)	Bomben and Sontheimer (2008); Shi et al. (2015)
	GBM (Glioblastoma multiforme)	A172	Inhibition of cell proliferation, migration and invasion	Inhibits TRPM7 channel	Leng et al. (2015)
	Nasopharyngeal carcinoma	CNE2, 5-8F, 6-10B	Inhibition of cell migration and invasion	Inhibits TRPC1 and TRPM7 channel	Chen et al. (2010); He et al. (2012)
		HK1	Inhibition of angiogenesis	Inhibits VEGF production and endothelial tube formation by blocking SOCE	Ye et al. (2018)
	Non-small-cell lung cancer (NSCLC)	H1975, A549, LLC-1	Inhibition of primary and metastatic Lewis lung cancer	Inhibits intracellular Ca^2+^mobilization	Qu et al. (2018)
BTP2	Colon cancer	HT29	Inhibition of cell growth	Inhibits SOCE channel and transcription cycling of topoisomerase-II α	Núñez et al. (2006)
	Cervical cancer	SiHa	Inhibition of cell migration	Inhibits actomyosin formation and cellular contractile force via blocking SOCE channel	Chen Y.-T. et al. (2013)
	Rhabdomyosarcoma (RMS)	RD, RH30	Inhibition of cell proliferation, migration and invasion	Inhibits SOCE channel	Schmid et al. (2016)
	Breast cancer	MDA-MB-468	Inhibition of cell proliferation, migration and invasion	Inhibits SOCE channel	Azimi et al. (2018)
	Prostate cancer	PC-3	Inhibition of cell invasion	Inhibits drebrin to bind to actin filaments	Dart et al. (2017)
Pyr3	triple negative breast cancer	MDA-MB-231	Inhibition of cell proliferation, inducement of apoptosis and cell death	Affects the TRPC3/RASA4/MAPK Pathway	Wang et al. (2019)
	Melanoma	C8161	Inhibition of cell proliferation and migration	Inhibits TRPC3 channel	Oda et al. (2017)
	Glioblastoma multiforme	LN229, U87	Inhibition of cell proliferation, migration and invasion	Dephosphorylates focal adhesion kinase and myosin light chain via inhibiting TRPC3 channel	Chang et al. (2018)
CAI	Melanoma	A2058	Inhibition of cell proliferation, adhesion and motility	Inhibits the receptor-mediated stimulation of effector enzymes	Kohn and Liotta (1990); Kohn et al. (1992)
			Inhibition growth and metastasis of transplanted xenografts	Inhibits calcium fluxes, arachidonic acid release, and phosphoinositides generation	
	Breast cancer	MDA-MB-231	Inhibition of cell proliferation, adhesion and motility	Inhibits the receptor-mediated stimulation of effector enzymes	Kohn and Liotta. (1990)
	Ovarian cancer	OVCAR-3	Inhibition of cell proliferation, adhesion and motility	Inhibits the receptor-mediated stimulation of effector enzymes	Kohn and Liotta (1990); Kohn et al. (1992)
			Inhibition growth and metastasis of transplanted xenografts	Inhibits calcium fluxes, arachidonic acid release, and phosphoinositides generation	
	Colon cancer	HT-29	Inhibition of the formation and growth of experimental pulmonary metastases in nude mice	Inhibits calcium fluxes, arachidonic acid release, and phosphoinositides generation	Kohn et al. (1992)
	Prostate cancer	DU-145, PPC-1, PC3, LNCaP	Inhibition of cell proliferation	Inhibits calcium fluxes and PSA production	Wasilenko et al. (1996)
	Glioblastoma	A172, T98G, U87, H4, U251	Inhibition of cell proliferation and adhesion	Inhibits calcium fluxes and calcium-sensitive signal transduction pathways	Jacobs et al. (1997)
	Head and neck squamous cell carcinoma	EVSCC14/17M, EVSCC19M, EVSCC18, UMSCC10A, FaDu	Inhibition of cell growth and invasion	Inhibits calcium fluxes	Wu et al. (1997)
	Hepatoma	Hep G2, Huh-7	Inhibition of cell proliferation	Inhibits calcium entry	Enfissi et al. (2004)
	Small cell lung cancer	NCI-H209, NCI- H345	Inhibition of cell growth, inhibition of xenograft proliferation in nude mice	Inhibits calcium fluxes	Moody et al. (2003)
				Inhibits the angiogenesis	
	Chronic myeloid leukemia	32D P210, 32D E255K	Inhibition of cell growth and decrease of BCR-ABL	Inhibits calcium influxes and signal transduction	Corrado et al. (2011)
	Liver metastases from B16F1 melanoma	B16F1	Decrease of the volume size and angiogenesis of tumor	Reduces the number of microvessels/mm^2^ and microvessel size	Luzzi et al. (1998)
	Lewis lung carcinoma	LLC cell	Inhibition of cell growth	Inhibits production of pro-inflammatory cytokines in TAMs	Ju et al. (2012)
	Melanoma	OVA-B16	Inhibition of tumor growth	Activates the CD8^+^ T cells and stimulates IDO1-Kyn metabolic circuitry	Shi et al. (2019)
RP4010	Esophageal cancer	KYSE-30, KYSE-150, KYSE-790, KYSE-190	Inhibition of cell proliferation and tumor growth	Inhibits SOCE channel-mediated Ca^2+^ oscillations	Cui et al. (2018)
	Pancreatic ductal adenocarcinoma	L3.6pl, BxPC-3, MiaPaCa-2	Inhibition of cell proliferation and colony formation	Inhibits CRAC channel	Khan et al. (2020)
ML-9	Pancreatic cancer	MIA PaCa-2, Panc1, BxPC3	Inhibition of cell invasion and adhesion	Inhibits MLCK to decrease the activity of cytoskeleton	Kaneko et al. (2002)
	Prostate cancer	LNCaP, PC3, DU-145	Inducement of prostate cell death	Stimulates autophagy via modulating Ca^2+^ homeostasis	Kondratskyi et al. (2014)
NPPB	Ovarian cancer	A2780	Inhibition of cell adhesion and invasion	Inhibits chloride channels leading to inhibition of [Ca^2+^]i	Li et al. (2009)
YZ129	Glioblastoma	U87	Inhibition of cell proliferation, migration and mobility	Inhibits calcineurin-NFAT pathway	Liu et al. (2019)
NSAIDs	Colon cancer	HT29	Inhibition of cell growth	Inhibits SOCE channel and COX-2 expression	Núñez et al. (2006); Wang et al. (2015)

## Store-Operated Calcium Entry-Targeted Compounds in the Application of Cancer Treatment

### SKF 96365

#### (1-[2-(4-Methoxyphenyl)-2-[3-(4-Methoxyphenyl)propoxy]ethyl]imidazole;hydrochloride)

SKF 96365 is structurally distinct from classic Ca^2+^ antagonists, drugs or compounds that inhibit Ca^2+^ entry by directly inhibiting Ca^2+^ channels or affecting Ca^2+^ pools. It shows selectivity in blocking receptor-mediated Ca^2+^ entry (RMCE), the Ca^2+^ influx that is independent of depolarization and caused by receptor occupation, leading to a significant increase in [Ca^2+^]i biologically, with no impact on internal Ca^2+^ release in platelets, neutrophils or endothelial cells (Spedding, 1982; Merritt et al., 1990; Cabello and Schilling, 1993), and the inhibition of RMCE exerted by SKF 96365 could inhibit [3H]-thymidine incorporation, interleukin-2 (IL-2) synthesis and cell proliferation of peripheral blood lymphocytes (Chung et al., 1994). It was also found that SKF 96365 could block store-independent TRP channels in osteoclasts and capacitative Ca^2+^ entry (CCE) in astrocytes (Wu et al., 1999; Bennett et al., 2001).

Iwamuro et al. found, for the first time, that SKF 96365 sensitively inhibited the SOCE activated by ET-1 at high concentrations (Iwamuro et al., 1999). As a SOCE inhibitor, SKF 96365 was reported to inhibit cell proliferation in many cancers by inducing cell apoptosis and cell cycle arrest in G2/M phase (Nordström et al., 1992; Lee et al., 1993; Arias-Montaño et al., 1998; Cai et al., 2009; Zeng et al., 2013; Zhang et al., 2015; Jing et al., 2016; Wang W. et al., 2018). Autophagy seems to play different roles in the pharmacological mechanism of SKF 96365. Inhibition of SOCE by SKF 96365 inhibited the AKT/mTOR pathway, induced autophagic cell death and decreased the survival, and proliferation of PC3 and DU145 prostate cancer cells (Selvaraj et al., 2016), while in colorectal cancer cells, SKF 96365 was reported to induce cytoprotective autophagy to delay apoptosis through the inhibition of calcium/calmodulin-dependent protein kinase IIγ (CaMKIIγ)/AKT signaling cascade, and autophagy inhibition could significantly augment the anticancer effect of SFK 96365 in mouse xenograft models (Jing et al., 2016).

In addition, SKF 96365 could also effectively inhibit the metastasis of cancers. It could impair the assembly and disassembly of focal adhesions of breast cancer cells (Yang et al., 2009), block the formation and activity of invadopodium in melanoma cells (Sun et al., 2014), and it could also inhibit cell migration by inactivating non-muscle myosin II and reducing actomyosin formation and contractile force in cervical and non-small cell lung cancer (NSCLC) cells (Chen Y.-T. et al., 2013; Wang Y. et al., 2018). In addition to inhibiting growth and colony formation independently through the blockage of Ca^2+^ channels, for example, through the blockage of TRPC channels in glioma cells (Bomben and Sontheimer, 2008; Ding et al., 2010; Song et al., 2014), SKF 96365 could also enhance the sensitivity of glioma cell lines to irradiation (Ding et al., 2010), suggesting that SKF 96365 may be developed not only as an anti-chemotherapy, but also as an adjuvant drug for radiotherapy.

In summary, the antineoplastic effects of SKF 96345 are universal. However, it has been confirmed that its effects are nonspecific (Franzius et al., 1994), and therefore it is necessary to conduct more studies to clearly delineate its specific mechanisms.

### 2-Aminoethoxydiphenyl Borate and Analogs

#### 2-APB (2-Diphenylboranyloxyethanamine)

Initial studies reported that 2-APB, as a novel membrane-penetrable modulator, inhibited Ca^2+^ release induced by IP_3_ without affecting IP_3_ binding to IP_3_R (IC_50_ = 42 µM) (Maruyama et al., 1997). Subsequently, 2-APB was found to be a reliable blocker of SOCE but an inconsistent inhibitor of IP3-induced Ca^2+^ release. With further study, it was found that 2-APB could exert both stimulatory and inhibitory effects on Ca^2+^ influx through CRAC channels: at low concentrations (1–5 µM), it activated SOCE pathway, while at high concentrations (≥10 µM), it blocked SOCE pathway completely (Prakriya and Lewis, 2001). The mechanisms of the dual effects of 2-APB on SOCE are also complicated: 2-APB could inhibit STIM1 directly by facilitating the coupling between CC1 (coiled-coil 1) and SOAR (STIM-ORAI-activating region) of STIM1. On the other hand, 2-APB could also impair the functions of STIM1 indirectly by interrupting the coupling between STIM1 and the mutant, ORAI1-V102C (Peinelt et al., 2008; Wei et al., 2016). Moreover, 2-APB could directly gate and dilate the pore diameter of ORAI1 and ORAI3 to regulate SOCE pathway without affecting STIM1 (DeHaven et al., 2008; Peinelt et al., 2008; Schindl et al., 2008; Yamashita et al., 2011; Amcheslavsky et al., 2014; Xu et al., 2016; Kappel et al., 2020). 2-APB also exerts multifaceted effects on transient receptor potential (TRP) channels (Colton and Zhu, 2007). It was reported that 2-APB could act as an agonist of TRPV1, TRPV2, TRPV3, TRPM6, and TRPC3 channels(Ma et al., 2003; Chung et al., 2004; Hu et al., 2004; Li et al., 2006), however, it exerted inhibitory roles on TRPC1, TRPC3, TRPC4, TRPC5, TRPC6, TRPC7, TRPM2, TRPM3, TRPM7, TRPM8, TRPV3, and TRPV6 channels (Hu et al., 2004; Poburko et al., 2004; Lievremont et al., 2005; Xu et al., 2005; Li et al., 2006; Bomben and Sontheimer, 2008; Togashi et al., 2008; Chokshi et al., 2012; Kovacs et al., 2012; Singh et al., 2018). Among these TRP channels, 2-APB could close the TRPV6 channel through protein–lipid interactions by binding to TRPV6 directly, and TRPV3 also was found to undergo similar structural changes triggered by 2-APB (Singh et al., 2018; Zubcevic et al., 2018).

When used as a blocker of SOCE at high concentrations, 2-APB displayed anticancer effects on rhabdomyosarcoma (RMS), nasopharyngeal carcinoma (NPC), prostate cancer, ovarian cancer, breast cancer, glioblastoma, lung cancer, melanoma, and cervical cancer (Bomben and Sontheimer, 2008; Chen Y.-T. et al., 2013; Zeng et al., 2013; Sun et al., 2014; Leng et al., 2015; Shi et al., 2015; Schmid et al., 2016; Sun et al., 2018; Liu et al., 2020). For instance, in breast cancer, 2-APB inhibited cell viability, and proliferation through inhibiting TRPM7 channel by arresting cell cycle in S phase not by promoting cell death (Liu et al., 2020). In melanoma and cervical cancer, 2-APB could reduce migration and invasion by inhibiting actomyosin formation, invadopodium assembly and maturation, through mechanisms similar to those of SKF 96365 action (Chen Y.-T. et al., 2013; Sun et al., 2014). 2-APB in NPC could also attenuate adhesive and invasive abilities by inhibiting TRPC1 and TRPM7 (Chen et al., 2010; He et al., 2012). Furthermore, it was reported that 2-APB could effectively antagonize the angiogenesis of NPC *in vivo* by inhibiting VEGF production and endothelial tube formation through the blockage of SOCE (Ye et al., 2018). In another study, 2-APB sensitized NSCLC cells to the antitumor effect of bortezomib (BZM) via suppression of Ca^2+^-mediated autophagy (Qu et al., 2018).

These effects suggest that 2-APB is attractive as a potentially potent therapy for primary cancer and metastatic cancer.

### 2-Aminoethoxydiphenyl Borate and Analogs

As 2-APB is a promising but not entirely specific SOCE inhibitor, Goto et al. explored two novel 2-APB structurally isomeric analogs in order to develop more specific and potent SOCE inhibitors: DPB-162AE and DPB-163AE (Goto et al., 2010). These two diphenylborinate (DPB) compounds are 100-fold more potent than 2-APB, and they are able to inhibit the clustering of STIM1 and block the ORAI1 or ORAI2 activity induced by STIM1 by inactivating the SOAR domain in STIM1. In particular, DPB-162 AE could consistently inhibit endogenous SOCE regardless of whether the concentration was high or low and exerted little effect on L-type Ca^2+^ channels, TRPC channels, or Ca^2+^ pumps when exerting maximal inhibitory effect on Ca^2+^ entry (Goto et al., 2010; Hendron et al., 2014; Bittremieux et al., 2017). However, the actions of DPB-163AE are more complex, showing a similar pattern to 2-APB by activating SOCE at low concentrations and inhibiting SOCE at higher levels (Goto et al., 2010).

Moreover, similar to 2-APB, at low concentrations (∼100 nM), both DPB-162AE and DPB-163AE could facilitate Orai3 currents, and at high concentrations (>300 nM), they transiently activated ORAI3 currents and then deactivated them. DPB compounds have been proven to activate ORAI3 in a STIM1-dependent manner, but they could not change the pore diameter of ORAI3, which is different from the mechanisms of 2-APB. It is speculated that because they are larger than 2-APB, DPB compounds are unable to enter the pore of ORAI3 (Goto et al., 2010; Hendron et al., 2014). In addition, DPB-162AE was reported to provoke leakage of Ca^2+^ from the ER into the cytosol in HeLa and SU-DHL-4 cells at concentrations required for adequate SOCE inhibition (Hendron et al., 2014; Bittremieux et al., 2017).

Although there have been no studies on DPB compounds with respect to cancer treatment to date, considering the specific inhibition of SOCE, DPB compounds are expected to be developed as potential anticancer drugs.

### Pyrazole Derivatives

#### Pyr2 (N-[4-[3,5-Bis(trifluoromethyl)pyrazol-1-yl]phenyl]-4-Methylthiadiazole-5-Carboxamide)

Pyr2, also known as BTP2 or YM-58483, was initially found to be able to inhibit SOCE, leading to impaired IL-2 production and NFAT dephosphorylation in Jurkat cells without affecting the T cell receptor (TCR) signal transduction cascade (Ishikawa et al., 2003). BTP2 also showed complicated effects on TRP channels, TRPC1, TRPC3, TRPC5, and TRPC6 channels were inhibited effectively; however, TRPM4 was activated by BTP2 at low concentrations in a dose-dependent manner. BTP-mediated facilitation of TRPM4, which is a Ca^2+^-activated cation channel that decreases Ca^2+^ influx by depolarizing lymphocytes, is the main mechanism for the suppression of cytokine release. Furthermore, it has been reported that the mechanism of inhibiting TRP channels, such as TRPC3 and TRPC5, involved in reducing their open probability rather than changing their pore properties without affecting the other Ca^2+^ signals in T cells including Ca^2+^ pumps, mitochondrial Ca^2+^ signaling and ER Ca^2+^ release (Zitt et al., 2004; He et al., 2005; Schwarz et al., 2006; Takezawa et al., 2006; Oláh et al., 2011; Wu et al., 2017).

BTP2 has exhibited inhibitory effects on several types of allergic inflammation, including autoimmune and antigen induced diseases through the suppression of cytokine release (IL-2, IL-4, IL-5, TNF-α, and IFN-γ) and T cell proliferation (Ohga et al., 2008; Law et al., 2011; Geng et al., 2012). Although many studies have indicated that BTP2 affects cancer through the modulation of immune cells, previous reports have mainly focused on the direct inhibition of cell proliferation, migration, and invasion of cancer cells themselves. For example, in colon cancer, BTP2 obviously decreased cell growth through direct SOCE inhibition (Núñez et al., 2006). BTP2 could also inhibit cell migration of cervical cancer, rhabdomyosarcoma (RMS), and breast cancer via blockage of SOCE (Chen Y.-T. et al., 2013; Schmid et al., 2016; Azimi et al., 2018); furthermore, the inhibition of cell migration in cervical cancer was due to the inhibition of actomyosin reorganization and contraction forces, similar to the effects of SKF96365 and 2-APB (Chen Y.-T. et al., 2013). It was also found that BTP2 could inhibit the proliferation and tubulogenesis of endothelial progenitor cells (EPCs), which are essential for the vascularization and metastatic switching of solid tumors (Dragoni et al., 2011; Lodola et al., 2012). On the other hand, BTP2 could inhibit the invasion of prostate cancer cells by impeding the binding of drebrin to actin filaments, with a SOCE independent mechanism (Dart et al., 2017).

In summary, the mechanism and effect of BTP2 on cancer are multi-aspect, and it is necessary to carry out further research on them for clarification.

#### Pyr3 (Ethyl1-(4-(2,3,3-Trichloroacrylamido)phenyl)-5-(Trifluoromethyl)-1h-Pyrazole-4-Carboxylate)

Pyr3 has mainly been recognized for directly and selectively inhibiting TRPC3 with attenuated activation of Ca^2+^-dependent signaling pathways, and structure-function relationship studies showed that the trichloroacrylic amide group is important for the TRPC3 selectivity of Pyr3 (Kiyonaka et al., 2009). The blockade of TRPC3-mediated Ca^2+^ signaling pathways by Pyr3 reduced cell proliferation, induced cell apoptosis and sensitized cell death to chemotherapeutic agents in triple-negative breast cancer through the inhibition of TRPC3-Ras GTPase-activating protein 4 (RASA4)-MAPK signaling cascade (Wang et al., 2019). In melanoma, Pyr3 also decreased the cell proliferation and migration *in vitro* and inhibited tumor growth *in vivo* by inhibiting TRPC3 and its downstream JAK/STAT5 and AKT pathways (Oda et al., 2017). Chang et al. reported that Pyr3 could inhibit the migration and invasion of glioblastoma multiforme (GBM) cells and reduce the size of tumor xenografts significantly by dephosphorylating focal adhesion kinase and myosin light chain (Chang et al., 2018). Subsequently, Pyr3 was found to effectively inhibit ORAI1-mediated SOCE in HEK293 cells and mast cells (RBL-2H3) in a dose dependent manner, and the amid-bond linked side-group pivotal for TRPC subtype selectivity was also proposed as a potential structural determinant for the SOCE inhibitory action (Schleifer et al., 2012).

#### Pyr6 and Pyr10 (N-[4-[3,5-Bis(trifluoromethyl)pyrazol-1-yl]phenyl]-3-Fluoropyridine-4-Carboxamide), (N-[4-[3,5-Bis(trifluoromethyl)pyrazol-1-yl]phenyl]-4-Methylbenzenesulfonamide)

It was reported that Pyr6 could inhibit both ORAI1-mediated SOCE and TRPC3 channels in mast cells leading to the suppression of mast cell activation, however, its effect on SOCE inhibition was 37-fold times that of TRPC3 inhibition, and it differed from the effect of Pyr10, which could specifically inhibit TRPC3 and had no effect on SOCE (Schleifer et al., 2012; Chauvet et al., 2016), thereby suggesting that Pyr6 and Pyr10 can be used as valuable tools to distinguish SOCE and TRPC3 channels.

#### GSK-5503A, GSK-7975A and GSK-5498A (2,6-Difluoro-N-[1-[(2-Phenoxyphenyl)methyl]pyrazol-3-yl]benzamide), (2,6-Difluoro-N-[1-[[4-Hydroxy-2-(trifluoromethyl)phenyl]methyl]pyrazol-3-yl]benzamide), (2,6-Difluoro-N-[1-[[2-Fluoro-6-(trifluoromethyl)phenyl]methyl]pyrazol-3-yl]benzamide)

Several novel pyrazole compounds including GSK-5503A, GSK-7975A, and GSK-5498A have been developed by GlaxoSmithKline as specific blockers of SOCE. GSK-5503A and GSK-7975A could inhibit STIM1-mediated ORAI1 and ORAI3 currents potentially via an allosteric effect on the selectivity filter of ORAI with a slow onset of action that did not have effects on STIM1-STIM1 oligomerization or STIM1-ORAI1 coupling (Yamashita et al., 2007; Derler et al., 2013). GSK-7975A could also efficiently inhibit the TRPV6 channel, possibly due to its architectural similarities to the selectivity filters of ORAI channels (Owsianik et al., 2006; Derler et al., 2013; Jairaman and Prakriya, 2013). GSK-5498A and GSK-7975A have been used to inhibit mediator and cytokine release from mast cells and T cells (such as IFN-γ and IL2) in multiple human and rat preparations by completely inhibiting SOCE (Rice et al., 2013). Although the roles of immune cells and cytokines are complicated in the tumor microenvironment, these compounds are expected to be applied as cancer treatments that function through anticancer immunity processes under certain circumstances.

#### Synta66 (N-[4-(2,5-Dimethoxyphenyl)phenyl]-3-Fluoropyridine-4-Carboxamide)

Synta66, also known as GSK1349571A, has garnered extensive attention in recent years because of its ability to selectively inhibit CRAC channels without affecting on PDGF- or ATP-evoked Ca^2+^ release, overexpressed TRPC5 channels, native TRPC1/5-containing channels, STIM1 clustering or nonselective store-operated cationic currents (Li et al., 2011). It has been confirmed that the potency of SOCE inhibition is directed against Orai1 in the order of Synta66 > 2-APB > GSK-7975A > SKF96365 > MRS1845 in human platelets (van Kruchten et al., 2012). By inhibiting SOCE effectively and specifically, Synta66 could inhibit the receptor-triggered mutual activation between Syk activation and Ca^2+^ influx in the RBL mast cell line, reduce the release of histamine, leukotriene C_4_ (LTC_4_), and cytokines (such as IL-5, IL-8, IL-13, and TNF-α) in human lung mast cells (HLMCs), and inhibit the expression of T-bet and the production of IL-2, IL17, and IFN-γ in lamina propria mononuclear cells (LPMCs) and biopsy specimens obtained from inflammatory bowel disease (IBD) patients (Ng et al., 2008; Di Sabatino et al., 2009; Ashmole et al., 2012). Azimi et al. compared the pharmacological inhibitory effects of Synta66 and BTP2 on SOCE pathway in breast cancer cell lines. They found that both Synta66 and BTP2 could inhibit the protease activated receptor 2 (PAR2) activator, and trypsin and EGF produced Ca^2+^ influx and serum-activated migration of MDA-MB-468 cells (Azimi et al., 2018). However, interestingly, Synta66, but not BTP2, had no effect on proliferation or EGF-activated cell migration, which are realized through unexplored mechanisms (Azimi et al., 2018). To date, no study has investigated whether Synta66 has anti-tumor effects in other cancers, nevertheless, Synta66 still has great potential to be developed as an available therapy for tumor treatment due to its specific and effective inhibition of SOCE.

### Monoclonal Antibodies

#### Anti-ORAI1 Monoclonal Antibodies

Lin et al. developed high-affinity fully human mAbs to human ORAI1, that bind to amino acid residues 210–217 of the human ORAI1 extracellular loop 2 domain (ECL2). These mAbs potently inhibited the SOCE, NFAT translocation and cytokine secretion from Jurkat T cells and in human whole blood (Lin et al., 2013). Another mAb to human native ORAI1, generated by Cox, also binds to ECL2 and could block the function of T cells both *in vitro* and *in vivo*, including the inhibition of T cell proliferation and cytokine production in immune cells isolated from rheumatoid arthritis patients and showed efficacy on an anti-ORAI1 human T cell-mediated graft-versus host disease (GvHD) mouse model (Cox et al., 2013), suggesting that mAb may be a novel treatment for humans with autoimmune diseases. Taken together, since anti-ORAI1 mAbs could impact on the autoimmune response, we speculate that they also show great potential to be used as cancer therapy by modulating of the tumor immune microenvironment.

### Inhibitors in Clinical Trials

#### Carboxyamidotriazole (5-Amino-1-[[3,5-Dichloro-4-(4-Chlorobenzoyl)phenyl]methyl]triazole-4-Carboxamide)

CAI (carboxyamidotriazole), also called L-651582, is one of the SOCE inhibitors that has been evaluated in clinical trials. It was initially developed as a coccidiostat and was confirmed to inhibit calcium influx, including SOCE, RMCE and VDCE (Hupe et al., 1991; Kohn et al., 1994; Sjaastad et al., 1996). Through the inhibition of Ca^2+^ influx and related signaling processes, such as receptor-associated tyrosine phosphorylation, arachidonic acid generation, and nucleotide biosynthesis, CAI displayed effective anticancer effects, including antiproliferative, antimigratory, antiangiogenic and antimetastatic properties, in a broad range of human tumors, including melanoma, glioblastoma, head and neck squamous cell carcinoma (HNSCC), hepatoma, small cell lung cancer (SCLC), chronic myelogenous leukemia (CML), and ovarian, breast, colon and prostate cancer (Kohn and Liotta, 1990; Kohn et al., 1992; Wasilenko et al., 1996; Jacobs et al., 1997; Wu et al., 1997; Luzzi et al., 1998; Moody et al., 2003; Enfissi et al., 2004; Corrado et al., 2011). Moreover, CAI could exert its anti-tumor activity through modulation of the tumor immune microenvironment by inhibiting proinflammatory cytokine production (such as TNF-α) in tumor-associated macrophages (TAMs), promoting IFN-γ release from activated CD8^+^ T cells or stimulating the IDO1-Kyn metabolic circuitry (Ju et al., 2012; Shi et al., 2019).

As a potential anticancer drug, CAI has been tested in phase I/II/III clinical trials, some of which showing that CAI could stabilize and improve the condition of patients with pancreaticobiliary carcinomas, melanoma, non-small cell lung cancer, epithelial ovarian cancer, prostate cancer, glioblastoma and other anaplastic gliomas while exhibiting a limited toxicity profile (Figg et al., 1995; Kohn et al., 1996; Bauer et al., 1999; Hussain et al., 2003; Hillman et al., 2010; Das, 2018; Omuro et al., 2018). However, its performance was barely satisfactory in many clinical trials, preventing it from being a first-line chemotherapy drug (Stadler et al., 2005).

#### RP4010

RP4010 was developed by Rhizen Pharmaceuticals. It was confirmed to block SOCE and SOCE-mediated Ca^2+^ oscillations in a dose-dependent manner, leading to the inhibition of NF-κB/p65 translocation to nuclei, thus impeding the proliferation and in ESCC xenograft tumor growth (Cui et al., 2018). In pancreatic ductal adenocarcinoma, RP4010 inhibited cancer cell proliferation and colony formation by reducing Akt/mTOR and Ca^2+^ influx-mediated NFAT signaling. Furthermore, RP4010 combined with gemcitabine and nab-paclitaxel could enhance anticancer activities in PDAC cells and patient-derived xenografts, indicating the potential of RP4010 as an anticancer chemotherapy (Khan et al., 2020). Indeed, RP4010 is in phase I/IB clinical trials currently.

#### CM4620 (N-[5-(6-Chloro-2,2-Difluoro-1,3-Benzodioxol-5-yl)pyrazin-2-yl]-2-Fluoro-6-Methylbenzamide)

CM4620, also called CM-128, was developed by CalciMedica and tested in phase I/II clinical trials to reduce pancreatitis. It could inhibit cell death pathway activation by SOCE inhibition in pancreatic acinar cells, thus leading to markedly reduced acute pancreatitis in mouse models (Wen et al., 2015). A recent study further demonstrated that in acute pancreatitis, CM4620 could not only inhibit the necrosis of parenchymal pancreatic acinar cells but could also prevent the activation of immune cells to reduce inflammation (Waldron et al., 2019). Although the effect of CM4620 on cancer cells is still unknown, it has the potential to be developed as a cancer therapy through its regulation of tumor immune cells.

### Other Small Molecular Inhibitors

ML-9 (1-(5-chloronaphthalen-1-yl)sulfonyl-1,4-diazepane) was initially described as an inhibitor of myosin light chain kinases (MLCKs) that binds at or near the ATP-binding site at the active center of kinases with or without Ca^2+^ calmodulin (Saitoh et al., 1987). Shortly thereafter, ML-9 was found to inhibit agonist-stimulated Ca^2+^ entry in endothelial cells without affecting the release of intracellular Ca^2+^ stores (Watanabe et al., 1996), and it was confirmed to inhibit SOCE in a dose-dependent manner by blocking rearrangement and puncta formation of STIM1 without inhibiting MLCKs. Thus far, ML-9 is the only inhibitor of STIM1 translocation (Smyth et al., 2008). Furthermore, ML-9 could also inhibit the activity of TRPC5 channel by impairing its plasma membrane localization through MLCK inhibition (Shimizu et al., 2006), causing apoptosis in both untransformed and transformed epithelial cells, retarding the growth of mammary and prostate cancer cells and blocking the invasion and adhesion of human pancreatic cancer cells (Kaneko et al., 2002). In another study, ML-9 reduced SOCE and induced Ca^2+^-dependent autophagy to promote the death of prostate cancer cells, but the effect of ML-9 on autophagy was independent of STIM1 and SOCE inhibition, suggesting that ML-9 may exert its effects on cancer cells through multiple mechanisms. Moreover, ML-9 combined with docetaxel could enhance the cell death of LNCaP, PC3 and DU-145 cells, suggesting that ML-9 may be developed as an adjuvant to anticancer chemotherapy (Kondratskyi et al., 2014).

NPPB (5-nitro-2-(3-phenylpropylamino)-benzoic acid), frequently used as a blocker of chloride channels, could also reduce CCE in endothelial cells (Gericke et al., 1994). It was also confirmed that NPPB could directly interact with CRAC to reversibly inhibit Ca^2+^ influx in Jurkat cells in a dose-dependent manner (Li et al., 2000). In ovarian cancer, NPPB could significantly inhibit A2780 cell adhesion and invasion (Li et al., 2009).

AnCoA4 (3-(6-methoxy-1,3-benzodioxol-5-yl)-8,8-dimethylpyrano[2,3-f]chromen-4-one) was identified by screening small-molecule microarray (SMM) using minimal functional domains of STIM1 and ORAI1, and it was found to inhibit Ca^2+^ influx by binding to the C-terminus of ORAI1 directly to perturb the interaction between STIM1 and ORAI1 by reducing the affinity of ORAI1 for STIM1. Through SOCE inhibition, AnCoA4 blocked T cell activation and inhibited the T cell-mediated immune response *in vitro* and *in vivo*, indicting the possibility that it can be used in therapeutic areas, including immunomodulation, inflammation and cancer (Sadaghiani et al., 2014).

RO2959 (2,6-difluoro-N-[5-[4-methyl-1-(1,3-thiazol-2-yl)-3,6-dihydro-2H-pyridin-5-yl]pyrazin-2-yl]benzamide) could specifically block SOCE by inhibiting the IP3-dependent CRAC current in native RBL-2H3 cells, CHO cells with STIM1/ORAI1 overexpression and human primary CD4^+^ T cells, resulting in a decrease in TCR-triggered gene expression, cell proliferation and cytokine production in T cells (Chen G. et al., 2013).

YZ129 (4-(isoquinolin-6-ylamino)-naphthalene-1,2-dione) was identified because of its inhibition of thapsigargin-triggered Ca^2+^ influx and NFAT nuclear entry through an automated high-content screening platform and it was found to exhibit potent anti-tumor activity against glioblastoma. It could bind to HSP90 directly and antagonize its calcineurin-chaperoning effect to reduce NFAT nuclear translocation and inhibit other key proto-oncogenic pathways, including hypoxic and glycolytic pathways and the PI3K/AKT/mTOR axis, thus leading to cell cycle arrest in G2/M phase of glioblastoma and promoting apoptosis and inhibition of tumor cell proliferation and migration (Liu et al., 2019).

MRS-1844 (DHPs-32) and MRS-1845 (DHPs-35) are compounds in the 1,4-dihydropyridine (DHP) family, and they were confirmed to inhibit store-operated calcium channels and reduce voltage-dependent L-type calcium channels. In addition, their potency in SOCE inhibition was much smaller than that of common SOCE inhibitors (the reported order is Synta66 > 2-APB > GSK-7975A > SKF96365 > MRS1845) (Harper et al., 2003; van Kruchten et al., 2012).

### Repurposing FDA-Approved Drugs

In addition to the abovementioned compounds targeting SOCE, other compounds that have been approved for clinical use in corresponding diseases were confirmed to inhibit SOCE. The ability of these compounds to inhibit Ca^2+^ influx through SOCE indicates the importance of learning more about their pharmacological properties and mechanisms, and the potential to extend their clinical indications.

Diethylstilbestrol (DES, 4-[(E)-4-(4-hydroxyphenyl)-hex-3-en-3-yl]-phenol) is a potent synthetic estrogen used for estrogen therapy in prostate and breast cancer. It was reported to inhibit SOCE and Ca^2+^ influx in a variety of cell types without affecting the whole-cell monovalent cation current mediated by TRPM7 channels. Trans-stilbene, a close structural analog of DES that lacks hydroxyl and ethyl groups, had no effect on CRAC current, further suggesting the specificity of DES for SOCE (Zakharov et al., 2004; Ohana et al., 2009; Dobrydneva et al., 2010).

Nonsteroidal anti-inflammatory drugs (NSAIDs) are commonly used to relieve pain, fever and inflammation. Epidemiological studies worldwide have demonstrated that NSAIDs also have cancer-protective effects, as these drugs are associated with a reduced risk of various types of cancer. In colon cancer, it has been reported that NSAIDs could exert antiproliferative effects through SOCE inhibition, and salicylate, the main aspirin metabolite, is considered a mild mitochondrial uncoupler that prevents mitochondrial Ca^2+^ uptake and promotes the Ca^2+^-dependent inactivation of SOCE, thus inhibiting the proliferation of HT29 cells (Núñez et al., 2006). Another study suggested that STIM1 overexpression promoted colorectal cancer progression through an increase in the expression of the pro-inflammatory and pro-metastatic enzyme cyclooxygenase-2 (COX-2). The inhibition of COX-2 with two NSAIDs, ibuprofen and indomethacin, abrogated STIM1-induced colorectal cancer (CRC) progression (Wang et al., 2015).

Mibefradil ([(1S,2S)-2-[2-[3-(1H-benzimidazol-2-yl)propyl-methylamino]ethyl]-6-fluoro-1-propan-2-yl-3,4-dihydro-1H-naphthalen-2-yl]2-methoxyacetate), a T-type Ca^2+^ channel blocker that was initially developed as a cardiovascular drug, was recently found to inhibit SOCE by blocking ORAI channels in a dose-dependent and reversible manner to significantly inhibit cell proliferation, induce cell apoptosis and arrest cell cycle in S and G2/M phases in HEK-293T-REx cells (Li et al., 2019).

## Conclusion and Perspectives

Ca^2+^ signaling is involved in almost all cellular activities in organisms. As a major route of Ca^2+^ entry in mammalian cells for replenishing the depleted intracellular Ca^2+^ store, SOCE regulates a diverse array of biological processes. Accumulating evidence has shown that STIM/ORAI-mediated SOCE is excessive in cancer tissues, and it is becoming clear that augmented SOCE promotes the malignant behavior of cancer cells, including tumor growth, angiogenesis, and metastasis. Therefore, SOCE could be a potential therapeutic target for the treatment of cancer.

As we summarized above, multiple compounds targeting SOCE have been developed and their efficiency in the inhibition of proliferation and migration of cancer cells has been evaluated. However, rare SOCE inhibitors have been approved for clinical use in cancer treatment due to their poor selectivity, which urgently needs to addressed. In addition, as SOCE is not the only channel for Ca^2+^ entry, cells could adopt other ways to obtain sufficient Ca^2+^ even after complete SOCE inhibition. Under this condition, drug combinations could be considered. Furthermore, due to the universal roles of Ca^2+^ signaling in cells, the cytotoxicity of SOCE inhibitors on normal cells and some anticancer immune cell inhibitors should also be considered in clinical anticancer applications. Developing SOCE inhibitors that could specifically target tumor tissues in certain circumstances is a hopeful therapeutic orientation toward cancer in the future.
